# Neuromuscular Electrical Stimulation Plus Rehabilitative Exercise as a Treatment for Dysphagia in Stroke and Non-Stroke Patients in an NHS Setting: Feasibility and Outcomes

**DOI:** 10.3390/geriatrics4040053

**Published:** 2019-09-24

**Authors:** Nicola Martindale, John Stephenson, Sue Pownall

**Affiliations:** 1 Sheffield Teaching Hospitals NHS Foundation Trust; Royal Hallamshire Hospital, Glossop Road, Sheffield S10 2JF, UK; 2 University of Huddersfield, Queensgate, Huddersfield HD1 3DH, UK; J.Stephenson@hud.ac.uk

**Keywords:** dysphagia, stroke, rehabilitation, neuromuscular electrical stimulation, NMES, Ampcare ESP

## Abstract

Dysphagia is a debilitating condition with significant consequences in terms of physical and mental health. This study demonstrates that it is feasible to provide an intensive therapy program combining neuromuscular electrical stimulation (NMES) with exercise against resistance in the treatment of dysphagia in a public healthcare setting. Thirty-one patients (17 stroke, 14 non-stroke) who experienced dysphagia with reduced laryngeal elevation completed the therapy program. After checking the data sets for comparability, it was deemed appropriate for the outcome data from these patients to be combined with that of 12 stroke patients previously reported to enable statistical analysis on a larger data set (n = 43). A repeated-measures ANOVA revealed a statistically significant increase in amount and variety of food a patient was able to take orally (FOIS) following completion of treatment (*p* < 0.001). There was no significant between-subject effect of stroke status (*p* = 0.43), or interaction between treatment and stroke status (*p* = 0.68). There was a significant improvement in secondary outcome measures of swallow safety with fluids (PAS) (*p* < 0.001) and swallow-related quality of life (Swal-Qol (*p* < 0.001). These findings indicate that the therapy program may be associated with reduced impairment in a subset of patients with dysphagia resulting from stroke and non-stroke atiologies, and the data will inform the design of future research to address unanswered questions.

## 1. Introduction

The use of neuromuscular electrical stimulation (NMES) in the treatment of dysphagia has received considerable research interest (see the National Institute for Clinical Excellence (NICE) guidelines, 2018, for an overview [1]). Dysphagia, or difficulty moving material from the mouth to the stomach, is a debilitating condition with far-reaching consequences in terms of a patient’s physical health [2,3], mental wellbeing [4], level of dependency and economic cost to the state [2,5]. Dysphagia can have many causes, including stroke, neurodegenerative conditions, traumatic brain injury and head and neck cancers. Severity of dysphagia also varies widely, from some people having a mild difficulty that may result in the avoidance of certain foods or eating situations; to those with severe cases, rendering a patient unable to safely eat or drink orally at all, thus requiring consideration of clinically assisted artificial nutrition and hydration. Standard management of dysphagia in the UK may involve exercise regimens, but is often restricted to symptom management through modification of oral intake and postural adaptations rather than direct rehabilitation of the swallow. There is a drive to pursue a more rehabilitative approach to dysphagia management to improve patient outcomes and reduce the economic burden of this patient group [6,7]. 

Transcutaneous NMES involves the application of electrical stimulation through the surface of the skin to the peripheral nerve fibers below, causing depolarization and corresponding muscle contraction [8]. The aim of NMES in the management of dysphagia is to increase the effectiveness of an individual’s swallowing ability by strengthening the muscles used in swallowing, and to promote the recovery of the cortical control of swallowing [9]. There is an increasing body of evidence to suggest that the use of NMES alongside traditional therapy exercises is beneficial, particularly for those patients who experience dysphagia following a stroke [1,10]. 

Swallowing is a complex activity, involving the coordinated action of oral, pharyngeal and laryngeal muscles [11] to ensure that the bolus is passed safely to the stomach, whilst the airway remains protected. Careful consideration must be given to which of these muscles are targeted with NMES to optimize functional gains. Many patients with dysphagia exhibit reduced laryngeal elevation during the swallow [12], which increases their risk of penetration and aspiration as their airway protection is compromised [13]. Incomplete laryngeal elevation also results in reduced cricopharyngeal sphincter opening, again impacting the effectiveness and safety of the swallow. The suprahyoid muscles are a group of four muscles which act to elevate the hyoid bone, and consequently the attached larynx, and are, therefore, an appropriate target for NMES aimed at improving swallow function [14]. The Ampcare effective swallowing protocol (ESP^TM^) is a therapy program which combines NMES to the suprahyoid muscles with the principles of repetitive, resistive exercise to promote neuroplasticity and optimize the outcomes of swallow therapy. It should be noted that laryngeal elevation is complex and also requires contraction of the longitudinal pharyngeal muscles [15], which are not targeted in this intervention. An additional requirement for airway protection is complete laryngeal vestibule closure, and to achieve full closure, complete contact of the arytenoids to the base of the epiglottis and full epiglottic inversion over the base of the arytenoids is required [16]. This process is not solely a biomechanical effect of laryngeal elevation and is achieved via different muscle groups; which are not targeted in this therapy program.

A pilot randomised control trial (RCT) exploring the efficacy of Ampcare ESP^TM^ found that patients with dysphagia following stroke had more positive outcomes in terms of improvements in functional oral intake and swallow-related quality of life if they completed this therapy program than if they had been randomized to the standard treatment group [6]. The improvement in swallowing and swallow-related quality of life—and the difference between the 2 groups—persisted at a one-month follow-up evaluation. Whilst these results are encouraging, larger-scale studies are required to further investigate the potential of NMES therapy programs in the treatment of dysphagia and to determine which population groups are most likely to benefit from the protocol and which element(s) of the program are required to see positive outcomes. Before conducting the necessary RCTs to address these important questions, however, it would be prudent to ensure that time-intensive NMES therapy programs can be offered widely within the constraints of NHS resources. 

In this study, local hospital charitable funding was secured to facilitate provision of the Ampcare ESP^TM^ NMES therapy program within a large teaching NHS hospital in the North of England, and to measure outcomes in accordance with National Institute for Health and Care Excellence (NICE) guidelines (2014) [17]. The therapy program was offered to patients with dysphagia resulting from stroke and non-stroke etiologies to determine whether the therapy program was effective, and whether there was a difference in response between stroke and non-stroke patients. Flexibility was afforded in the delivery of treatment, for example, sessions were offered fewer than five times a week when necessary, depending on therapist and patient availability, and exercise programs were tailored to each patient, depending on their dysphagia presentation and ability to follow instructions. This approach was adopted to maximize compliance within a real-world clinical setting and to determine whether this impacted on the outcomes. 

The aims of the current work were to (1) determine whether it is possible to provide an intensive NMES therapy program within a publicly funded Speech and Language Therapy service, and (2) to combine the new outcome data generated (new data) with that previously published [6] to (a) allow meaningful statistical analysis on the efficacy of the therapy and (b) to inform the design of future RCTs to further investigate the value of NMES therapy programs in the treatment of dysphagia. 

## 2. Materials and Methods

A single-arm trial was conducted to gather new outcome data for stroke and non-stroke patients with dysphagia who completed the NMES therapy program within a publicly funded health care system. A purposive sampling strategy was employed, such that all patients who were identified as having reduced laryngeal elevation and who fulfilled the inclusion criteria were offered the NMES therapy program (Ampcare ESP^TM^). Reduced elevation was determined during videofluoroscopy examination by perceptual judgement that there was reduced or partial superior movement of the thyroid cartilage, resulting in partial approximation of the arytenoids to the epiglottic petiole. Ratings were made independently by two speech and language therapists who both had over 15-years’ experience of completing videofluoroscopy examinations. Although a standardized rating scale was not employed, reliability testing had been confirmed by a process of peer review.

For those patients who consented to the treatment, outcome measures were collected at baseline and after completion of the therapy program by experienced SLTs involved in the patients’ care. This new data was combined with previously published outcome data [6] to enable meaningful statistical analysis on the efficacy of the therapy and to inform the design of future RCTs. 

### 2.1. Selection Criteria

#### 2.1.1. Inclusion

Patients were offered the NMES therapy program if they:

- Experienced dysphagia with reduced laryngeal elevation (confirmed by video fluoroscopy examination).
- Were able to generate a swallow—either to command or reflexively—with the use of a lollipop to stimulate saliva production.
- Were able to commit to regular SLT sessions (20 in total).

In contrast to Sproson et al. 2018 [6], who specified that patients had to be at least one-month post-stroke, no limit was set regarding time since the onset of dysphagia. 

#### 2.1.2. Exclusion

Participants were excluded if they were:

- Under 18 years of age.
- Had a pacemaker or other serious cardiac disease.
- Had lesions/infections to the treatment site.
- Had severe cognitive difficulties (at a level where they were unable to follow simple instructions).

### 2.2. Outcome Measures

In line with the NICE guidelines for the use of NMES in the treatment of dysphagia (2014) [17], and consistent with Sproson et al. 2018 [6], the following outcome measures were used to monitor changes in the safety of the swallow:

- Functional oral intake scale (FOIS, [18])—validated measure of the dependence of a patient on artificial nutrition/hydration and the amount and variety of food taken orally.
- Penetration–Aspiration Scale (PAS, [19])—scale used to rate the presence and severity of penetration/aspiration of contrast during video fluoroscopy. The video fluoroscopy assessments used a frame rate of 30 frames per second and followed a standard protocol, but were necessarily guided by clinical judgement and patient safety; therefore, it was not appropriate for all patients to attempt the same foods and drinks. The protocol included the use of a standardized recipe to mix barium with the stimuli and a standardized protocol for the presentation of each of the stimuli to the individual. This ranged from thin barium to solid textures (sandwich and biscuit) and included standardized volumes of each food and drink assessed, e.g., teaspoon of fluid through to cup drinking and measured amounts of solid boluses presented. Where possible individuals were encouraged to self-feed, however, support with eating/drinking was offered if required. To allow comparisons within subjects, the PAS score selected for analysis was that for the same quantity and viscosity of fluid at baseline and on completion of the therapy program.
- Swallow-Related Quality of Life (Swal-Qol, [20])—questionnaire to capture patients’ ratings of domains of dysphagia and their impact on quality of life (QoL).

### 2.3. Intervention

Treatment sessions took approximately 45 minutes, including set-up and time to pack away and debrief with the patient. Each patient was offered 20 treatment sessions which were completed in 4–7.5 weeks, depending on patient and therapist availability. This differed from Sproson et al. (2018) [6], whereby therapy sessions were completed in 4 weeks. All therapy sessions were conducted under the guidance of an Ampcare ESP^TM^ trained SLT in the most appropriate place for the patient. In-patients received the therapy at their bedside, or in a quiet room within the hospital. Out-patients were invited to clinic if they were able to travel. If this was not feasible, therapy appointments were provided within the patient’s own home, or residential/nursing home.

**Figure 1:**
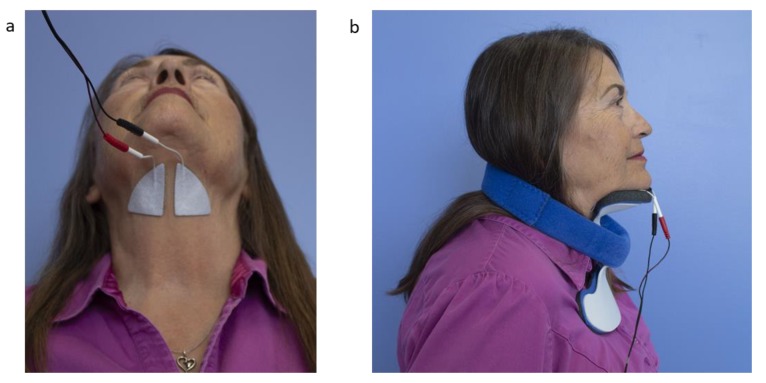
Photograph to illustrate the Ampcare ESP^TM^ equipment. Electrode placement is show in (**a**) and intervention set up, including the postural device used to provide resistance shown in (**b**). *Patient provided written consent for the photographs to be published.

Ampcare ESP^TM^ includes the use of NMES delivered to the submental region, specifically targeting the suprahyoid muscles to facilitate laryngeal elevation. Electrodes were positioned superior to the thyroid notch. See Figure 1 for illustration of the electrode placement and set-up of equipment. Muscle contraction was observed in the majority of patients, enabling verification that the stimulation resulted in hyolaryngeal elevation. If muscle contraction was not visible, palpation was used to confirm electrode placement. In a minority of cases, it was not possible to detect muscle contraction, and therapy proceeded providing the patient reported feeling the sensation of stimulation. In no instance was hyolaryngeal depression observed. See Table 1 for full details of the stimulation parameters, which were consistent with those used in Sproson et al. 2018 [6].

A pulse rate of 30 Hz was used, providing muscle contraction without fatigue or muscle spasm. The cycle-on time lasted for 5 seconds. Initially, cycle-off time was set at 25 seconds, resulting in 60 swallow attempts per session. Once the patient was reliably generating swallows on each stimulation, the cycle-off period was reduced on the subsequent session to 20 seconds. Finally, if the patient was able, and consented to do so, the cycle-off period was reduced to 15 seconds, producing 90 swallow attempts per session. The intensity of electrical stimulation was initially increased until the patient reported feeling a tingling sensation. They were then asked, ‘Can you tolerate more?’ at regular intervals during the 30-minute treatment time to facilitate a gradual increase in pulse intensity, within personal tolerance levels. 

During stimulation, patients were instructed to complete a specific swallow exercise. In contrast to Sproson et al., 2018 [6], where each patient followed the same exercise regimen, the exercise program in this study was customized for each patient, depending on their pattern of impairment identified at video fluoroscopy, and their ability to follow instructions. The patient was asked to complete the same exercise for a block of 10 minutes. They were then given a short break before continuing with the therapy, attempting a different exercise, according to their individual therapy program (see Table 2 for the range of exercises employed). Patients were encouraged to complete all exercises against resistance, by pushing down into a postural neck brace support (see Figure 1b) as it has been shown that completing a chin to chest exercise against resistance results in activation of hyolaryngeal musculature [21]. Patients who were unable to follow more complex instructions were encouraged to attempt an effortful swallow with chin to chest against resistance on each stimulation, using a lollipop if required to assist saliva generation. A stopwatch was used to provide a verbal prompt 5 seconds before the onset of the electrical stimulation where necessary, to maximize the chance of the patient swallowing in sync with stimulation.

### 2.4. Analysis

Descriptive data from stroke patients were compared between new and old data sets to verify that there was no evidence for systematic differences in the two samples and that the two data sets could be meaningfully combined and considered as a single data set of stroke patients. Data compatibility was assessed both in terms of a comparison of mean values and frequencies; and in terms of the numbers of patients experiencing either improvement, deterioration or no change in measured outcomes.

Following verification of data compatibility in stroke patients, new data gathered in this study (17 stroke patients and 14 non-stroke patients) were combined with that of the 12 stroke patients with complete data sets who completed the NMES therapy program (Ampcare ESP^TM^) as part of Sproson et al.’s (2018) pilot RCT in the evaluation of the effect of treatment [6] (resulting in a total of 43 patients). This augmented data set was summarized both as a complete cohort and according to stroke status (i.e., stroke and non-stroke patients). The extent and nature of any missing data was evaluated.

The primary within-participants outcome variable was functional oral intake score (FOIS). Secondary within-participants outcome variables were the penetration/aspiration score for fluids (PAS) and Swal-Qol score. Each outcome variable was measured pre- and post-treatment. Post-treatment videofluoroscopy was undertaken within two weeks of completion of the therapy program for all but one patient, whose videofluroscopy was delayed as the patient was unwell (unrelated to dysphagia)

The main between-participants variable was *Stroke status*; categorized as Stroke or Non-Stroke. Other between-participants variables recorded for descriptive purposes included: age in years (*Age*), gender (*Gender*), time in months from onset of dysphagia to start of therapy (*Onset-treatment*), number of completed therapy sessions (*Sessions*), time in weeks to complete therapy (*Treatment duration*) and whether or not a postural device was used (*Postural devic*e). 

Participants who suffered significant medical deterioration during the treatment process were noted. Parallel analyses were conducted on the full sample, and on those patients who did not suffer medical deterioration during the treatment process only, as a sensitivity study.

A repeated measures analysis of variance (RM-ANOVA) was conducted on the data, using pre-and post-treatment scores relating to the primary outcome of FOIS. Stroke status was included as the key between-participants variable. The sample size precluded the inclusion of further between-participants variables in the analysis to avoid model over-fitting. Significance levels of main effects and interactions were used to test hypotheses of parallelism, flatness and equality of levels. Marginal means of each outcome measure were determined pre- and post-treatment, with pairwise comparisons assessed for significance using Bonferroni-corrected *p*-values. Secondary outcomes were assessed using paired samples t-tests. Effect sizes, confidence intervals and significance levels were reported for all procedures.

### 2.5. Ethical Approval

Local ethical and governance approval was obtained from Sheffield Teaching Hospitals NHS Foundation Trust, in accord with NICE guidelines [17]. All treated patients gave informed written consent for completing the therapy program and for outcome measures to be collected and analyzed before beginning the therapy program.

## 3. Results

Thirty-two patients fulfilled the inclusion criteria (17 stroke, 15 non-stroke). All patients who were offered the NMES therapy program wished to take part. One non-stroke patient did not complete the therapy program as it became apparent that she was unable to reliably generate swallows during therapy sessions and she returned to standard dysphagia management within the SLT service. 

### 3.1. Descriptive Statistics

Patient characteristics and outcome measures from the study of Sproson et al. [6] and the new data are compared in Table 3 below. The patient samples are broadly comparable, with no substantive differences on any characteristic except time of onset to treatment; the values of which were considerably lower in the current data set. The inclusion criteria in Sproson et al. [6] specified patients must be more than one-month post-stroke at onset of therapy. This was not specified for the current study, however, only two of the patients began treatment less than one month after their stroke.

Further comparison of the new stroke data gathered in this study with that presented previously [6] showed similar proportions of stroke participants who experienced improvement, deterioration or no change in each of the outcome measures (Table 4). Just under 60% of stroke patients who completed the therapy program were able to eat a more varied and complex diet at the end of their treatment as indicated by an increase in their FOIS score, just under 60% showed an improvement in their ability to swallow fluids safely, as evidenced by a decrease in their PAS score and almost 90% reported an improvement in their swallow-related quality of life, as indicated by an increase in their Swal-Qol score. Patients within the non-stroke group had a variety of diagnoses (see Table 5) and due to small numbers data were not analyzed according to diagnosis. A total of 43% of the non-stroke group had an improved FOIS following completion of the NMES therapy program, 64% were observed to have a safer swallow for fluids, as seen by a reduction in PAS score, and 62% reported an improvement in their swallow-related quality of life (Swal-Qol).

With the descriptive comparison of data sets revealing no evidence for a systematic difference between the two sets of stroke patients, the new data from this single-arm trial were combined with existing data for stroke patients who had completed the NMES therapy program in the same NHS Trust previously (n = 12; [6]). In total, the combined data set includes 43 patients aged 25–92 years: 32 males (74.4%) and 11 females (25.6%). The full sample is summarized descriptively in Table 6. Statistics are given in terms of mean (SD; range) for numerical variables, and frequency (valid %) for categorical variables.

Four patients in the new data set exhibited significant medical deterioration during the assessment period, these patients were all within the stroke group. In each case, the medical complication involved chest or urinary sepsis, requiring treatment with antibiotics. These patients continued with the NMES therapy program when they were well enough to engage with the therapy sessions.

No data was recorded as missing on the primary outcome measure (FOIS), or secondary outcome variable PAS fluids. The secondary outcome variable Swal-Qol data were incomplete for two patients. One non-stroke patient was unable to complete the assessment due to the language demands of the assessment, as they had a long-standing severe aphasia (language impairment). A second patient, who had a stroke diagnosis, completed the Swal-Qol before commencing the therapy program but was distressed at the end of the therapy due to her ongoing severe dysphagia, and, therefore, it was felt to be unethical to ask the patient to complete the Swal-Qol at this time. The data could be inferred to be missing at random from separate variance t-tests and the missing data were not imputed. 

### 3.2. Analysis of Primary Outcome

A repeated-measures ANOVA conducted on all patients (n = 43) revealed that in a model with stroke status as the between-participants variable, the main effect of treatment was significantly related at the 5% significance level to the primary outcome of FOIS (F_1,41_ = 22.6, *p* < 0.001). The substantive effect of Treatment was moderately large (partial-η^2^ = 0.355). The main effect of Stroke status, and the Treatment × Stroke status interaction, were not significant (F_1,41_ = 0.634, p = 0.430 for Stroke status; F_1,41_ = 0.174; *p* = 0.678 for the interaction). Hence, the hypothesis of flatness of profiles was rejected; the hypotheses of equal levels and parallelism of profiles were not rejected. 

**Figure 2:**
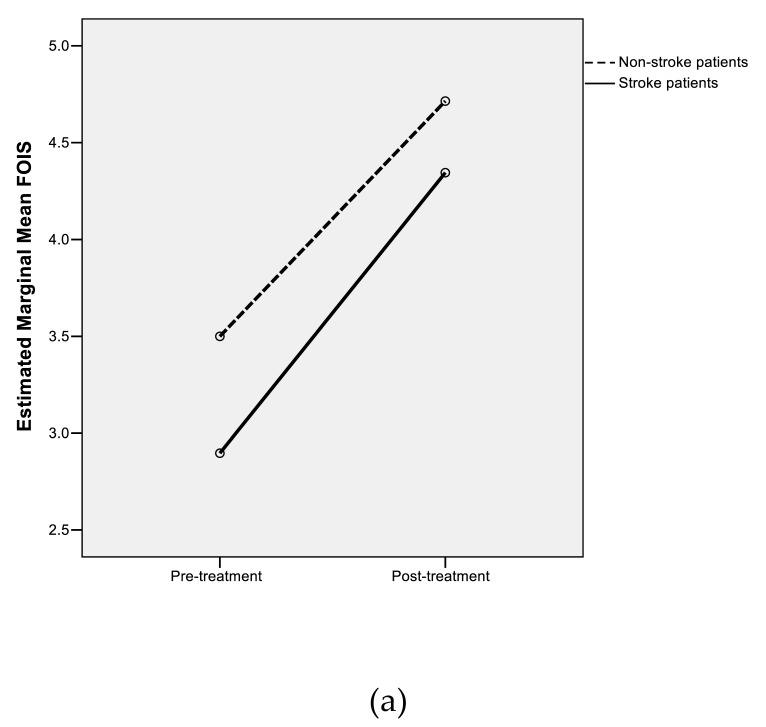

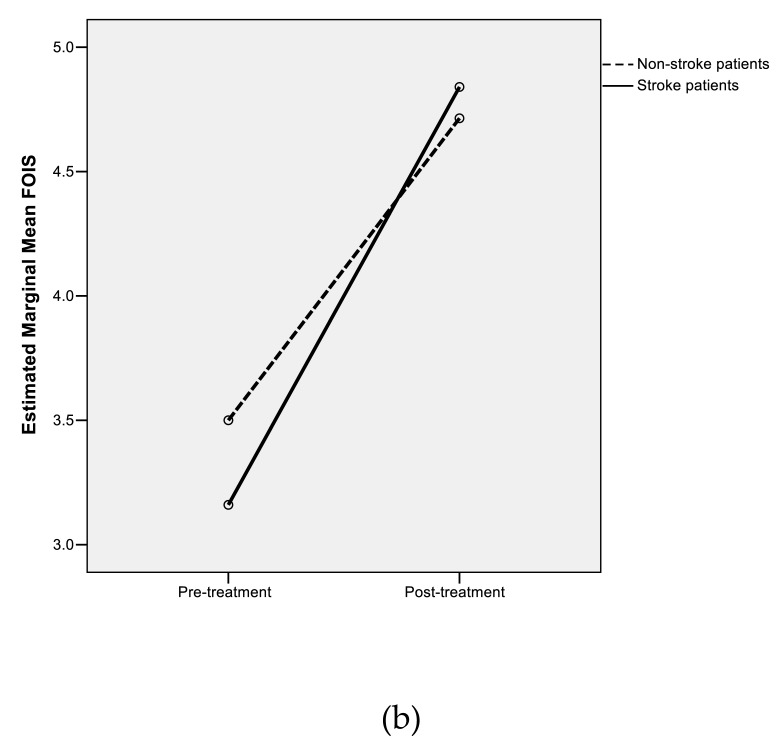
(**a**) Marginal means of FOIS for all stroke and non-stroke patients. (**b**) Marginal means of FOIS for stroke and non-stroke patients not exhibiting medical deterioration.

A parallel repeated-measures ANOVA conducted on all patients not suffering from medical deterioration (n = 39) revealed that in a model with Stroke status as the between-participants variable, the main effect of Treatment was significantly related at the 5% significance level to the primary outcome of FOIS (F_1,37_ = 25.3, *p* < 0.001). The substantive effect of Treatment was moderately large (partial-η^2^ = 0.406). The main effect of Stroke status, and the Treatment × Stroke status interaction, were not significant (F_1,37_ = 0.032, *p* = 0.858 for stroke status; F_1,37_ = 0.656; *p* = 0.424 for the interaction). Hence, the hypothesis of flatness of profiles was rejected; the hypotheses of equal levels and parallelism of profiles were not rejected. Marginal means of stroke and non-stroke patients are shown in Figure 2a (all patients) and Figure 2b (all patients not exhibiting medical deterioration).

Marginal mean FOIS scores of all outcomes measured pre- and post-treatment, with associated 95% confidence intervals and Bonferroni-corrected significance levels for each pairwise comparison, are summarized in Table 7.

The inclusion or exclusion of those patients who had suffered from medical deterioration during the treatment process did not have a substantive effect on the findings of the analysis, although a non-significant Treatment × Stroke status interaction can be observed in the analysis of patients who did not suffer from medical deterioration.

### 3.3. Analysis of Secondary Outcomes

A series of paired samples t-tests conducted on pre- and post-treatment secondary outcome data of all patients revealed that post-treatment scores were significantly different on *PAS (Fluids)* (*p* < 0.001) and *SwalQol* (*p* < 0.001) outcomes.

A corresponding series of paired samples *t*-tests conducted on pre- and post-treatment secondary outcome data of all patients who did not suffer from medical deterioration revealed that post-treatment scores were significantly different on *PAS (Fluids)* (*p* < 0.001) and *SwalQOL* (*p* < 0.001) outcomes.

In the context of secondary outcomes, Bonferroni corrections for multiple comparisons were not applied to either series of results. The significant differences were all of large magnitude according to Cohen’s *d* statistic, calculated using pooled standard deviations. 

*P*-values, differences of means and associated 95% confidence intervals (CIs) derived from (i) the full sample and (ii) patients not suffering from medical deterioration are summarized in Table 8 and Table 9.

### 3.4. Additional Findings

#### 3.4.1. Dependence on Artificial Feeding

Eight stroke patients and four non-stroke patients included in the study were unable to take any food or drink orally (i.e., were nil by mouth) and were artificially fed at the beginning of the treatment. After completion of the NMES therapy program, 5 of the 8 stroke patients (62.5%) and one of the non-stroke patients (25%) were able to safely introduce some level of oral intake. One of the non-stroke patients whose swallow did not recover sufficiently following the treatment to allow safe introduction of oral diet and fluids reported that “I know it (Ampcare ESP^TM^) has improved my swallowing, the exercises are easier. I can actually generate a swallow”. This patient continued to practice swallowing exercises (without the electrical stimulation or postural device) after completion of the NMES therapy program and his swallow improved, allowing him to introduce oral diet and fluids 3 months later. The other two non-stroke patients who were nil by mouth and did not experience a functional gain in their swallow after treatment both had dysphagia as a result of head and neck cancer and associated treatments.

**Figure 3:**
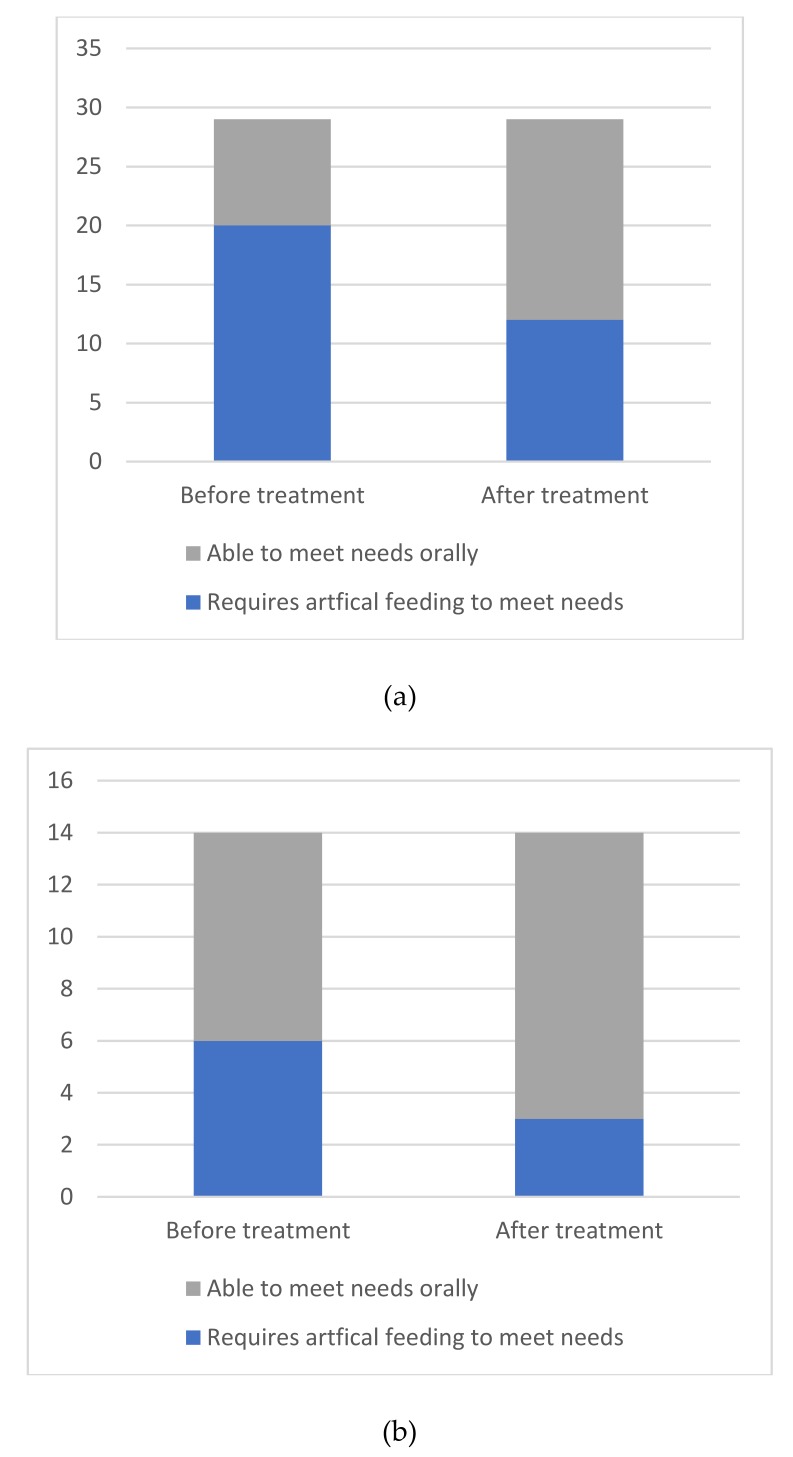
(**a**) Number of stroke patients dependent on artificial feeding before and after completion of the NMES therapy programme. (**b**) Number of non-stroke patients dependent on artificial feeding before and after completion of the NMES therapy programme.

Twenty-six patients included in this study were dependent on artificial nutrition to meet their nutritional, hydration and medication needs before completing the NMES therapy program; 20 with a stroke diagnosis and six with a non-stroke etiology. After completion of the therapy, eleven of these patients (eight stroke and three non-stroke) had recovered sufficiently to enable them to meet their needs orally, thus no longer requiring artificial nutrition and hydration (Figure 3a,b).

#### 3.4.2. Feedback from Patients

No significant adverse effects were reported during the course of the therapy by any of the patients. Even those patients whose swallow did not improve talked positively about the therapy; patients felt more informed about their swallowing and their difficulties and felt that they had done all that they could to try to improve this. Patients reported valuing the flexibility of service provision and the ability to access therapy within their own home. Patients were generally discharged from Speech and Language Therapy soon after completion of the therapy.

## 4. Discussion

This study has provided additional evidence that a proportion of patients with dysphagia relating to stroke and non-stroke etiologies who complete an NMES therapy program (Ampcare ESP^TM^) experience an improvement in their swallow. Amalgamation of the new data collected in the single-arm study with that previously published [6] revealed a significant and substantive positive change in all outcome measures, demonstrating an improvement in safety of the swallow for fluids, an increase in the quantity and variety of oral diet taken and an improvement of swallow-related quality of life post-treatment. It is noteworthy that studies of this size (n = 43) are rarely powered to reveal significant results, and, therefore, it is unusual to reveal significant findings, particularly of this magnitude. The large effect sizes derived in this study (Cohen’s *d* = 0.724 to 0.821) are consistent with synthesized estimates for overall effect derived in a systematic review and meta-analysis on the effect of NMES on swallowing rehabilitation [28], which revealed effect sizes as measured by Hedges’ *g* statistic of 0.62 to 0.66. This meta-analysis considered only clinical trials and, hence, the estimates of effect were based on standardized differences between independent treatment and control groups; in contrast with the analysis of the current study, in which subjects acted as their own controls. Although not all included studies used FOIS as a primary outcome, synthesised measures of effect were standardized accordingly: Hedge’s *g* statistic, as quoted by Carnaby-Mann and Crary [28], is calculated using weighted standard deviations, but is otherwise identical to Cohen’s statistic which uses unweighted values. 

Recently published NICE guidelines regarding the application of NMES in the treatment of dysphagia found insufficient evidence to support its use for non-stroke populations [1]. However, in the current study, the primary outcome scores in stroke and non-stroke patients were found to be similar, and the relative effect of the treatment was found to be similar in stroke and non-stroke patients. It is of note that two patients who did not show an improvement following the NMES therapy program had dysphagia resulting from head and neck cancer and associated treatments, in agreement with previous work in this area which has similarly found limited benefit for this patient group [29]. 

National stroke guidelines [30] suggest that the outcome of dysphagia research should focus on freedom from tube feeding, QOL and duration of treatment effect. Of the 26 people who were dependent on artificial feeding prior to treatment, 15 were able to safely meet their nutrition, hydration and medication needs orally, post-treatment. As may be expected, all of these patients who were able to return to oral feeding reported an improvement in their QoL as demonstrated by an increase in their Swal-Qol score. In addition, a further 17 patients reported an increase in their swallow-related QoL, and as such, nearly three-quarters of the patients who completed the treatment program benefited from an improvement in their QoL. Previous work has demonstrated that the improvements seen following completion of this NMES therapy program are sustained [6]. Patients in the current study were not formally followed-up after completion of their treatment; however, anecdotally, patients were reported to sustain, if not further improve, their swallow status after completion. The economic savings associated with reduced dependence on artificial feeding were not calculated in this study. This is an area that will require further investigation to allow consideration of the health economics of the therapy.

A significant limitation of this study as an evaluation of the efficacy of the NMES therapy program is that it is a single-arm design. The absence of a control group who did not receive the NMES therapy program limits the conclusions that can be drawn regarding the efficacy of the program as a treatment for dysphagia. It is not possible, for example, to control for the spontaneous recovery that is likely to contribute to the progress exhibited by patients with dysphagia as a consequence of stroke. However, it is of note that patients who had dysphagia as a result of non-stroke etiologies also improved. These patients would not be expected to show spontaneous recovery, and indeed some, including those with Parkinson’s disease, may be expected to deteriorate. This strengthens the hypothesis that the NMES therapy program is likely to have contributed to the degree and/or speed of recovery in the stroke group. Furthermore, the fact that the improvement in swallow outcome measures was evident in the stroke cohort even when stroke patients who experienced general deterioration in their medical condition were included suggests that the changes observed cannot be wholly attributed to spontaneous recovery. 

The assessment of the effectiveness of the Ampcare ESP^TM^ protocol by Sproson et al. [6], which recorded follow-up data in both control and treatment arms, reported an improvement in FOIS scores of 0.8 from a baseline of 4.3 (18.6%) for the control group who received standard therapy (i.e., not the NMES therapy program). The treatment group saw a larger improvement in FOIS of 1.6 from a baseline of 3.5 (45.7%) [6]. The improvement in FOIS scores for the stroke cohort in the current study, all of whom received the Ampcare ESP^TM^ therapy program, was 1.4 from a baseline of 2.5 (56%). These figures suggest that the improvement in FOIS observed in the current study is in line with that reported previously for the same intervention, and, furthermore, that the component of improvement that may be ascribed to reasons other than the direct effect of the intervention is relatively low. 

The time between onset of dysphagia and treatment was shorter in the new data set than Sproson et al. [6]. The proportion of patients who benefited from the treatment, however, was remarkably similar, indicating that the therapy may be beneficial for a proportion of patients with dysphagia after stroke, whether they suffered their stroke weeks, months or even years before commencing the therapy. Evidence suggests that in order to optimize recovery, it is likely to be beneficial to offer therapy as soon as possible post-stroke, to facilitate brain plasticity [31]. These findings highlight the need for further investigation into the potential of NMES therapy programs, and the analyses conducted in this study will help with the design of such research.

As this study was trialing the use of an NMES therapy program within a real-world clinical setting, the Speech and Language Therapists completing post-therapy outcome measures were not blinded to the fact that the patient had completed the therapy program, which may be considered a limitation. However, the therapists followed a rigorous video fluoroscopic protocol, to ensure accurate and objective PAS measures were recorded, and scores were recorded by two therapists to further increase validity and reliability. In future studies, it may be advantageous for an independent therapist who is not involved in the patient’s care to complete the Swal-Qol with the patient, however, this was not practical in the current study. Additionally, in this study, the inclusion criteria of reduced laryngeal elevation was rated from perceptual judgment and was thus subjectively assigned. Advances in digital measurements which can be utilized in videofluoroscopy analysis to accurately measure structural movements should be included in future studies to add rigour to patient selection and outcome measures.

This study was not designed to explore which of the components of the NMES therapy program are necessary to see an improvement in swallow outcome. Future RCTs will be required to facilitate the partitioning of the potential effects of NMES, use of postural device to provide resistance, swallow exercises conducted, and the intensity of the therapy program. What this study has demonstrated, however, is that NMES therapy programs provide a promising treatment approach to improve outcomes for a proportion of patients with dysphagia and may reduce the economic burden of this client group, and, importantly, that it can be delivered successfully within a public healthcare setting. Current National Stroke Guidelines recommend that “People with stroke should accumulate at least 45 minutes of each appropriate therapy every day, at a frequency that enables them to meet their rehabilitation goals, and for as long as they are willing and capable of participating and showing measurable benefit from treatment.” [30] (p52). It should, therefore, be feasible to provide NMES therapy programs requiring intensive delivery, for patients with dysphagia post-stroke. Furthermore, this study has demonstrated that it may not be necessary to provide the therapy every day for benefits to be observed, thus increasing the potential for its use with non-stroke populations who traditionally may not receive such intensive therapy in public healthcare settings. The findings, therefore, suggest that NMES therapy programs can be usefully applied to clinical settings within the NHS.

It is important to note that not everyone who completed the NMES therapy program in this study experienced improvements in their swallow function. The small sample size precludes the concurrent assessment of all patient-level covariates (age, gender, time between onset of symptoms and commencement of treatment, etc.). Further research is necessary to establish which populations are most likely to benefit from this therapy approach. 

The data from this study will be used to inform the design of future RCTs investigating the potential of NMES therapy programs as a treatment for dysphagia and to begin to address these unanswered questions. For example, data revealed that mean functional oral intake score (FOIS) in 29 stroke patients at baseline was 2.90 (SD 1.915) and 4.34 (SD 2.143) post-intervention; hence, the estimated difference between the two groups was 1.44 units. A sample size calculation determined that for a two-arm study with participants allocated to control or treatment in equal proportions, group sizes of 43 patients per group (i.e., 86 in total) would be required to achieve 90% power to reject the null hypothesis of equal means when the population mean difference is 1.44 units, with standard deviations of 1.915 and 2.143 for control and intervention groups, respectively, using a two-sided two-sample unequal-variance t-test. The Type I error probability associated with the test of the null hypothesis is 0.05. To allow for 15% attrition loss, a minimum of 102 patients would need to be recruited to the study.

## 5. Conclusions

The current study has demonstrated that the provision of intensive dysphagia therapy is possible within an NHS setting, particularly for the stroke population who are recommended to have 45 minutes therapy per day in national guidelines [30]. It has shown that the provision of an NMES therapy program (Ampcare ESP^TM^) resulted in significant and functional improvements in swallow safety and an increase in swallow-related quality of life in a subset of patients with dysphagia resulting from both stroke and non-stroke etiologies. Benefits of the intervention were observed in some patients who received the therapy less intensively, increasing the feasibility of providing it for non-stroke patients in a clinical setting. Future studies are required to explore the contribution of the individual active element(s) of the treatment protocol and the population of patients who are most likely to benefit from the intervention; the information presented here will help to guide the design of this research. 

## Figures and Tables

**Table 1:** Ampcare ESP^TM^ neuromuscular electrical stimulation (NMES) stimulation parameters.

Frequency (Hz)	30
Phase duration (µsec)	50
Ramp up (seconds)	1
Ramp down (seconds)	0
Cycle on time (seconds)	5
Cycle off time (seconds)	Initially 25, reduced to 20 and then 15 seconds if patient able to reliably generate a swallow during each ‘on-time’
Program duration (minutes)	30 (with breaks after each 10-minute block)
Intensity	0–20 depending on patients’ individual tolerance

**Table 2:** Swallow exercises with supporting evidence for their selection (table adapted from [6]).

**Exercise**	**Evidence**
Chin to chest against resistance with effortful swallow	Chin to chest against resistance produced more muscle activity than shaker head lift [21] Effortful swallow produces earlier onsets and peaks of pharyngeal pressures, greater driving force to move boluses through the pharynx and reduced pharyngeal residue [22]
Chin to chest against resistance with Mendelsohn	Mendelsohn maneuver improves laryngeal and hyoid elevation [23,24]
Chin to chest, followed by jaw opening and closing, followed by effortful swallow	Jaw opening against resistance produced significant improvements in hyolaryngeal movement and wider upper esophageal sphincter opening [25]
Chin to chest against resistant with Masako	Masako maneuver is performed to strengthen the contact between the tongue base and laryngo-pharyngeal wall [26,27]

**Table 3:** Comparative summary of stroke patients (new data *c.f.* data from Sproson et al., 2018 [6]).

Variable	Mean (SD; Range) or Frequency (valid %)		
	Stroke Patients from Sproson et al. (*n* = 12)	Stroke Patients from New Data Set (*n* = 17)	All Stroke Patients (*n* = 29)
Gender			
Male	8 (66.7%)	14 (82.4%)	22 (75.9%)
Female	4 (33.3%)	3 (17.6%)	7 (24.1%)
Age (years)	75.5 (11.4; 57–92)	71.7 (13.3; 41–88)	73.3 (12.5; 41–92)
Onset to treatment (months)	17.3 (25.0; 1–73)	1.7 (1.44; 0.5–4.0)	8.12 (17.5; 0.5–73)
Medical deterioration			
during therapy			
No	12 (100.0%)	13 (75.6%)	25 (86.2%)
Yes	0 (0.0%)	4 (24.4%)	4 (13.8%)
Number of therapy sessions	20.0 (0.00; 20–20)	18.8 (1.70; 15-20)	19.3 (1.42; 15–20)
Treatment duration (weeks)	4.29 (0.334; 4–5)	5.00 (0.866; 4–7.5)	4.71 (0.774; 4–7.5)
Device			
No	0 (0.0%)	1 (5.9%)	1 (3.4%)
Yes	12 (100.0%)	16 (94.1%)	28 (96.6%)
FOIS pre-treatment	3.42 (2.07; 1–7)	2.53 (1.78; 1–7)	2.90 (1.92; 1–7)
FOIS post-treatment	4.92 (1.98; 1–7)	3.94 (2.22; 1–7)	4.34 (2.14; 1–7)
PAS (fluids) pre-treatment	6.67 (1.92; 3–8)	5.12 (2.69; 1–8)	5.76 (2.49; 1–8)
PAS (fluids) post-treatment	4.33 (2.96; 1–8)	3.29 (2.76; 1–8)	3.72 (2.8; 1–8)
SwalQOL pre-treatment (n = 42)	103 (15.4; 82–134)	95.4 (32.7; 42–143)	98.5 (26.8; 42–143)
SwalQOL post-treatment (n = 41)	116 (14.7; 96–138)	125 (30.7; 54–157)	121.1 (5.1; 54–157)

**Table 4:** Patient outcomes shown separately for the new data set, previously reported data [6], and the combined dataset.

Outcome Measure	Improved		Deteriorated		No change	
	Stroke	Non-stroke	Stroke	Non-stroke	Stroke	Non-stroke
**FOIS**						
New data	10/17 (58.8%)	6/14 (42.9%)	1/17(5.8%)	0/14 (0%)	6/17 (35.3%)	8/14 (57.1%)
Sproson et al., 2018 data	7/12 (58.3%)		0/12 (0%)		5/12 (41.7%)	
Combined data	17/29 (58.6%)		1/29 (3.4%)		11/29 (37.9%)	
**PAS fluid**						
New data	10/17 (58.8%)		2/17 (11.8%)	2/14 (14.3%)	5/17 29.4%)	3/14 (21.4%)
Sproson et al., 2018 data	7/12 (58.3%)	9/14 (64.3%)	0/12 (0%)		5/12 (58.3%)	
Combined data	17/29 (58.6%)		2/29 (6.9%)		10/29 (34.4%)	
**Swal-Qol**						
New data	14/16 (87.5)	8/13 (61.5%)	2/16 (12.5%)		0/16 (0%)	
Sproson et al., 2018 data	10/12 (83.3%)		2/12 (16.7%)	4/13 (30.8%)	0/12 (0%)	1/13 (7.7%)
Combined data	24/28 (85.7)		4/28 (14.2%)		0/28 (0%)	

**Table 5:** Medical diagnoses of non-stroke patients.

Diagnosis	Number of Patients
Parkinson’s disease	2
Head and neck cancer	2
Traumatic Brain Injury	1
Repeated chest infections, unknown etiology	4
Left carotid endarterectomy	1
Thyroidectomy	1
Post critical care complications including voice and dysphagia	1
Necrotizing autoimmune myopathy	1
Benign essential tremor	1

**Table 6:** Descriptive summary of total sample (new data plus Sproson et al., 2018 [6]).

Variable	Mean (SD; range) or Frequency (valid %)		
	Non-Stroke Patients (*n* = 14)	Stroke Patients (*n* = 29)	All Patients (*n* = 43)
*Gender*			
Male	10 (71.4%)	22 (75.9%)	32 (74.4%)
Female	4 (28.6%)	7 (24.1%)	11 (25.6%)
*Age* (years)	67.8 (18.1; 25–87)	73.3 (12.5; 41–92)	71.5 (14.6; 25–92)
Onset to treatment (months)	24.9 (33.1; 2–96)	8.12 (17.5; 0.5–73)	13.6 (24.6; 0.5–96)
Medical deterioration during therapy			
No	14 (100.0%)	25 (86.2%)	39 (90.7%)
Yes	0 (0.0%)	4 (13.8%)	4 (9.3%)
Number of therapy sessions	19.5 (1.40; 15–20)	19.3 (1.42; 15–20)	19.5 (1.20; 15–20)
Treatment duration (weeks)	5.04 (0.909; 4–7)	4.71 (0.774; 4–7.5)	4.81 (0.824; 4-7.5)
Device			
No	3 (21.4%)	1 (3.4%)	4 (9.3%)
Yes	11 (78.6%)	28 (96.6%)	39 (90.7%)
FOIS pre-treatment	3.50 (2.10; 1–7)	2.90 (1.92; 1–7)	3.09 (1.97; 1–7)
FOIS post-treatment	4.71 (2.16; 1–7)	4.34 (2.14; 1–7)	4.47 (2.13; 1–7)
PAS (fluids) pre-treatment	5.36 (1.95; 1–8)	5.76 (2.49; 1–8)	5.63 (2.31; 1–8)
PAS (fluids) post-treatment	3.64 (2.65; 1–8)	3.72 (2.8; 1–8)	3.70 (2.75; 1–8)
SwalQOL pre-treatment (n = 42)	98.3 (23.1; 65–143)	98.5 (26.8; 42–143)	98.5 (25.5; 42–143)
SwalQOL post-treatment (n = 41)	109.6 (36.1; 49–161)	121.1 (5.1; 54–157)	117.5 (29.1; 49–161)

**Table 7:** Estimated marginal means of primary outcome measure pre- and post-treatment.

Variable	Timepoint	Marginal Mean	95% CI for Marginal Mean	Bonferroni-Corrected *p*-Value
FOIS ^1^	Pre-treatment	3.20	(2.55, 3.85)	<0.001
	Post-treatment	4.53	(3.82, 5.24)	
FOIS ^2^	Pre-treatment	3.33	(2.66, 4.00)	<0.001
	Post-treatment	4.78	(4.11, 5.45)	

^1^ All patients.^2^ All patients not exhibiting medical deterioration.

**Table 8:** Parameters from paired-samples testing: all patients.

Variable	Difference in Means ^1^	SD of Difference	95% CI for Difference	*t*-Value	Df	*p-*Value	Cohen’s *d*
*PAS (Fluids)*	−1.93	2.85	(−2.81, −1.05)	−4.44	42	<0.001	0.760
*SwalQOL*	19.8	25.3	(11.8, 27.7)	5.01	40	<0.001	0.724

^1^ Post-treatment – pre-treatment.

**Table 9:** Parameters from paired-samples testing: all patients not suffering from medical deterioration.

Variable	Difference in Means ^1^	SD of Difference	95% CI for Difference	*t*-Value	Df	*p-*Value	Cohen’s *d*
*PAS (Fluids)*	−2.03	2.63	(−2.88, −1.17)	−4.81	38	<0.001	0.821
*SwalQOL*	20.9	23.5	(13.0, 28.7)	5.38	36	<0.001	0.817

^1^ Post-treatment – pre-treatment.

## References

[1] National Institute for Health and Care Excellence (NICE) (2018). Transcutaneous Neuromuscular Electrical Stimulation for Oropharyngeal Dysphagia in Adults. https://www.nice.org.uk/guidance/ipg634.

[2] Smithard D.G., O’Neill P.A., Parks C., Morris J. (1996). Complications and outcome after acute stroke. Does dysphagia matter?. Stroke.

[3] Sura L., Madhavan A., Carnaby G., Crary M.A. (2012). Dysphagia in the elderly: Management and nutritional considerations. Clin. Interv. Aging.

[4] Chen P.H., Golub J.S., Hapner E.R., Johns M.M. (2009). Prevalence of perceived dysphagia and quality-of-life impairment in a geriatric population. Dysphagia.

[5] Altman K.W., Yu G.P., Schaefer S.D. (2010). Consequence of dysphagia in the hospitalized patient: Impact on prognosis and hospital resources. Arch. Otolaryngol. Head Neck Surg..

[6] Sproson L., Pownall S., Enderby P., Freeman J. (2018). Combined electrical stimulation and exercise for swallow rehabilitation post-stroke: A pilot randomized control trial. Int. J. Lang. Commun. Disord..

[7] Gallas S., Marie J.P., Leroi A.M., Verin E. (2010). Sensory Transcutaneous Electrical Stimulation Improves Post-Stroke Dysphagic Patients. Dysphagia.

[8] Huckabee M.L., Doeltgen S. (2007). Emerging modalities in dysphagia rehabilitation: Neuromuscular electrical stimulation. N. Z. Med. J..

[9] Pownall S., Enderby P., Sproson L., Majid A. (2017). Electrical Stimulation for the Treatment of Dysphagia. Electroceuticals: Advances in Electrostimulation Therapies.

[10] Chen Y.-W., Chang K.-H., Chen H.-C., Liang W.-M., Wang Y.-H., Lin Y.-N. (2016). The effects of surface neuromuscular stimulation on post-stroke dysphagia: A systematic review and meta-analysis. Clin. Rehabil..

[11] Perlman A.L., Palmer P.M., McCulloch T.M., Vandaele D.J. (1999). Electromyographic activity from human laryngeal, pharyngeal, and submental muscles during swallowing. J. Appl. Physiol..

[12] Kuhl V., Eicke B.M., Dieterich M., Urban P.P. (2003). Sonographic analysis of laryngeal elevation during swallowing. J. Neurol..

[13] Logemann J.A. (1988). Swallowing physiology and pathophysiology. Otolaryng. Clin. N. Am..

[14] Kim S.J., Han T.R. (2009). Effect of Surface Electrical Stimulation of Suprahyoid Muscles on Hyolaryngeal Movement. Neuromodulation.

[15] Pearson W.G., Taylor B.K., Blair J., Martin-Harris B. (2016). Computational analysis of swallowing mechanics underlying impaired epiglottic inversion. Laryngoscope.

[16] Vose A., Humbert I. (2019). “Hidden in plain sight”: A descriptive review of laryngeal vestibule closure. Dysphagia.

[17] National Institute for Health and Care Excellence (NICE) (2014). Transcutaneous Neuromuscular Electrical Stimulation for Oropharyngeal Dysphagia. https://www.nice.org.uk/guidance/ipg490.

[18] Crary M.A., Mann G.D., Groher M.E. (2005). Initial psychometric assessment of a functional oral intake scale for dysphagia in stroke patients. Arch. Phys. Med. Rehabil..

[19] Rosenbek J.C., Robbins J.A., Roecker E.B., Coyle J.L., Wood J.L. (1996). Penetration-aspiration scale. Dysphagia.

[20] McHorney C.A., Robbins J., Lomax K., Rosenbek J.C., Chignell K., Kramer A.E., Bricker D.E. (2002). The Swal-Qol and Swal-Care outcomes tool for oropharyngeal dysphagia in adults: III. Document of reliability and validity. Dysphagia.

[21] Watts C.R. (2013). Measurement of hyolaryngeal muscle activation using surface electromyography for comparison of two rehabilitative dysphagia exercises. Arch. Phys. Med. Rehabil..

[22] Burnett T.A., Mann E.A., Cornall S.A., Ludlow C. (2003). Laryngeal elevation achieved by neuromuscular stimulation at rest. J. Appl. Physiol..

[23] Lazarus C., Logemann J.A., Song C.W., Rademaker A.W., Kahrilas P. (2002). Effects of voluntary maneuvers on tongue base function for swallowing. Folia Phoniatr. Logop..

[24] McCullough G.H., Kim Y. (2013). Effects of the Mendelsohn manoeuvre on extent of hyoid movement and UES opening post-stroke dysphagia. Dysphagia.

[25] Wada S., Tohara H., Iida T., Inoue M., Sato M., UEDA K. (2012). Jaw-opening exercise for insufficient opening of upper oesophageal sphincter. Arch. Phys. Med. Rehabil..

[26] Fujiu M., Logemann J.A. (1996). Effect of a Tongue-Holding Maneuver on Posterior Pharyngeal Wall Movement During Deglutition. Am. J. Speech Lang. Pathol..

[27] Pisegna J., Langmore S. (2014). The Efficacy of the Masako (Tongue-Hold) Maneuver: A Pilot Study. https://open.bu.edu/handle/2144/12190.

[28] Carnaby-Mann G.D., Crary M.A. (2007). Examining the Evidence on Neuromuscular Electrical Stimulation for Swallowing. A meta analysis. Arch. Otolaryngol. Head Neck Surg.

[29] Langmore S.E., Mccullock T.M., Krisciunas G.P., Lazarus C.L., Van Daele D.J., Pauloski B.R., Rybin D., Doros G. (2016). Efficacy of electrical stimulation and exercise for dysphagia in patients with head and neck cancer: A randomized clinical trial. Head Neck..

[30] Rudd A.G., Bowen A., Young G., James M.A. (2016). National Clinical Guideline for Stroke.

[31] Hara Y. (2015). Brain Plasticity and Rehabilitation in Stroke Patients. J. Nippon Med. Sch..

